# Spina bifida and cardiorespiratory profile: the impact of leisure sport activities on physical fitness

**DOI:** 10.1007/s00381-023-06152-3

**Published:** 2023-09-09

**Authors:** Riccardo Monti, Francesco Mariani, Rosanna Mastricci, Francesco Maria Nifosì, Vincenzo Palmieri, Ester Manes Gravina, Margherita Capriati, Claudia Rendeli

**Affiliations:** 1 Sports Medicine and Functional Re-Education Centre, Department of Ageing, Orthopaedic and Rheumatological Sciences, “Agostino Gemelli” University Polyclinic Foundation – IRCCS, Rome, Italy; 2 Department of Women’s and Children’s Health Sciences and Public Health, “Agostino Gemelli” University Polyclinic Foundation – IRCCS, Rome, Italy; 3 Cognitive-Functional Unit, Department of Ageing, Orthopaedic and Rheumatological Sciences, “Agostino Gemelli” University Polyclinic Foundation – IRCCS, Rome, Italy; 4 Spina Bifida and Malformative Uropathies Centre, Department of Women’s and Children’s Health Sciences and Public Health, “Agostino Gemelli” University Polyclinic Foundation – IRCCS, Rome, Italy

**Keywords:** Spinal dysraphism, Sports activity, Cardiorespiratory function, Cardiopulmonary Exercise Test, Bone mineral density

## Abstract

**Purpose:**

The aim of the present study is to evaluate a population of young patients affected by Spina Bifida (SB) to describe their cardiorespiratory function and bone mineral density profile, analyzing any differences between people performing and those who do not perform sports activity. The study also aimed to rule out possible congenital heart disease associated with spina bifida, considering the common origin of certain cardiac structures with those found to be altered in SB patients.

**Methods:**

Thirty-four young patients, aged between 12 and 22 years, diagnosed with spinal dysraphism (SD), have been clinically described and, in order to evaluate their physical fitness, functional capacity and bone mass, almost all of them underwent a complete cardiorespiratory assessment, including electrocardiogram (ECG), echocardiogram, Cardiopulmonary Exercise Test (CPET), body composition analysis using bioimpedance analysis (BIA) and Dual Energy X-ray Absorptiometry (DEXA), as well as the estimation of bone mineral density (BMD) with Computerized Bone Mineralometry (CBM).

**Results:**

Collected data demonstrated that only 35% of the subjects practiced physical activity during the week. BMI and percentage FM values were pathological in at least 50% of the population. On cardiological investigations (ECG and echocardiogram), no significant alterations were found. In all patients who performed CPET (79.4%), pathological values of the main functional capacity parameters were revealed, especially peak oxygen consumption (VO_2_ peak), even when corrected for BCM or FFM estimated at BIA and DEXA, respectively. In the CBM analysis, out of 27 patients in whom the femoral *T*-score was evaluated, a condition of osteopenia was revealed in 40.7% of the patients (11/27) and osteoporosis in 18.5% (5/27); out of 27 patients in whom the lumbar *T*-score was evaluated, 37% of the patients showed osteopenia (10/27) and 29.6% osteoporosis (8/27). When the comparison between exercising and non-exercising patients was performed, the only statistically significant difference that emerged was the median lumbar *T*-score value, which appeared lower in the group not performing physical activity (*p* = 0,009).

**Conclusions:**

The extensive cardiorespiratory evaluation, including CPET, of our cohort of spina bifida patients showed altered values of the main parameters related to cardiorespiratory fitness and is the only study in the literature that analysed bone mineralization values in physically active and sedentary spina bifida patients and demonstrated a statistically significant difference. Furthermore, it is the only study to date that investigated the possible association of congenital heart diseases with SD, without demonstrating the existence of pathological conditions.

## Introduction

Spinal dysraphism (SD), also called spina bifida (SB), is a general term that includes a spectrum of congenital multifactorial abnormalities derived from a maldevelopment of the ectodermal, mesodermal and neuroectodermal tissues between 17 and 30 days of fetal development [1–3]. The severity of the disorder can range from completely asymptomatic to functionally impaired [4]. The commonest and severe form is the myelomeningocele (MMC), characterized by a dorsally opened spinal cord [3]; other conditions, such as myeloschisis, meningocele, lipomeningomyelocele, and tethered cord, may be usually less severe and are part of the closed type of SD, also called “occult.” This condition also correlates with cutaneous alterations, including hemangiomas, hypertrichosis, pigmentary nevi, pendulous fibroma, lipomas, dermal sinuses, and gluteal sulcus deviation [5]. These children also present Arnold-Chiari malformation (hypoplasia of the posterior cranial fossa and cerebellum, with associated hydrocephalus from obstruction of cerebrospinal fluid flow at the level of the third and fourth cerebral ventricles), paraplegia, “neurogenic” bowel and bladder, and, in some cases, hydrocephalus [6]. Furthermore, the 50% of them develop urinary tract infections (UTIs) [7] during the first 15 months of life [8]. In addition, it is important to remember that, during the third week of embryonic life, an ectodermal-derived structure called the “neural crest” differentiates, whose cells originate from the dorsolateral region of the neural tube, which in the case of spina bifida sufferers, as already mentioned, manifests a closure defect.

Depending on the type and level of the lesion, impairments related to deficits in cognition, motor function, and sensory function may arise [9, 10]. A systematic review conduct in 2014 provided a critical synthesis of physical fitness components and effects of exercise training on these components in individuals with SB: patients with SB have impaired cardiorespiratory endurance, muscle strength, body composition, and flexibility compared with healthy peers, and cardiorespiratory endurance and muscle strength components seem to improve with exercise training [11]. Poor cardiorespiratory endurance in childhood has been described as having important consequences for the development of cardiovascular disease in later life [12]. Moreover, moderate and high levels of cardiorespiratory fitness have been associated with lower risk of mortality from all-causes regardless of gender, age, and body composition [13]. Therefore, to prevent cardiovascular diseases and reduce mortality rates, it is crucial to enhance cardiorespiratory endurance in individuals with SB since early childhood. Furthermore, children and adolescents with spina bifida who use a manual wheelchair are less physically active and more sedentary than typically developing youth [14]; therefore, in addition to the main pathology, they are associated with a series of other co-morbidities that are the inevitable consequence of forced inactivity.

Aim of this study is to describe the cardiorespiratory characteristics of our cohort of patients with spinal dysraphism to evaluate any eventual alteration. Moreover, given the common origin of part of the neural tube with certain cardiovascular structures, as mentioned above, our research group considered it reasonable to carry out a cardiological study to rule out possible congenital heart diseases, such as abnormal origins of the coronary arteries or congenital cardiac outflow tract anomalies (conotruncal heart malformations), in the pediatric spina bifida patient population. These conditions should always be ruled out because they represent a contraindication to the practice of sporting activity, including leisure ones.

## Methods

In the period between May 2022 and April 2023, we recruited patients afferent to the spina bifida and Malformative Uropathies Centre of the “Agostino Gemelli” University Polyclinic Foundation – IRCCS in Rome, in order to better assess cardiorespiratory function and bone mineral density profile.

The study protocol included:

- A thorough medical-sports evaluation including the collection of a detailed medical history, including sports history, objective examination with collection of anthropometric measurements. In ambulatory subjects in wheelchairs, height was obtained by summing the measurements of individual body segments (length of head from vertex to chin, length of neck from chin to suprasternal point, length of thorax from suprasternal point to pubic symphysis, length of lower limb from anterosuperior iliac spine to internal malleolar point, distance from malleolar point to internal heel). The measurement was made with a flexible tape measure. Weight was measured with a Seca 635 platform scale (Seca Inc., CA, USA), initially measuring the weight of the patient sitting in their wheelchair and then subtracting the weight of the wheelchair alone.
- Twelve-lead resting electrocardiogram (Cardioline ECG200S, Cardioline S.p.A., Trento, Italy).
- Two-dimensional echocardiography (Toshiba Aplio Artida SSH-880 CV, Toshiba Medical Systems, Inc. Tustin, CA, USA). Echocardiographic measurement of left atrial diameter (LA), right ventricular end-diastolic diameter (RVEDD), left ventricular end-diastolic diameter (LVEDD), left ventricular end-systolic diameter (LVESD), interventricular septum end-diastolic thickness (IVSd), and posterior wall end-diastolic thickness (PWd) were performed in parasternal long-axis view; left ventricle ejection fraction (EF) was calculated in 4 chambers view using the Simpson’s single plane algorithm; the diameter of the inferior vena cava (IVC) was measured from the subcostal view. For verifying the absence of diastolic dysfunction, the mitral E/A ratio was measured in the 4-chambers view.
- Cardiopulmonary Exercise Test (CPET) (or, if not possible, a classic exercise stress test) with an arm-crank ergometer (Ergoline Ergoselect 400, ergoline GmbH, Bitz, Germany and K5 Metabolimeter, COSMED The Metabolic Company SRL, Rome, Italy), using an incremental exercise protocol of 10 W every minute. Simultaneous ECG detection was performed by “Mortara X-Scribe” cardiac stress test detector connected to a “Mortara X12+ ” wireless transmitter (Mortara Instrument Europe S.r.l., Casalecchio di Reno, Bologna, Italy).
- Bioelectrical Impedance Analysis (BIA; Akern Bodygram Plus, Akern SRL, Pontassieve, Florence, Italy) with analysis of body cellular mass (BCM), fat mass (FM), fat free mass (FFM), relative FFM (expressed as a percentage of the whole body mass), relative FM (expressed as a percentage of the whole body mass), and total body water (TBW). The BIA was performed placing four gel electrodes on the dorsal surfaces of the right hand and foot, with a test frequency of 50 kHz.
- Computerized Bone Mineralometry (CBM) for the measurement of bone mineral density (BMD) and, therefore, to assess the presence of osteopenia and osteoporosis by detection of the *T*-score, considering both the *T*-score value related to the femoral head and that of the lumbar spine. This analysis was, in addition, combined with the Dual Energy X-ray Absorptiometry (DEXA) technique to study the body composition of the patients by detecting fat mass and lean mass (sum of body mass cell and fat free mass) values of each.

The examination was carried out using “Hologic Discovery DXA” bone densitometer and the results analyzed using “Hologic Discovery DXA System” software (Hologic Inc., Mississauga, Ontario, Canada).

### Statistical analysis

For continuous variables the Kolmogorov-Smirnov test was used to assess whether the distribution was normal or not. Categorical variables were reported as count and percentage. Continuous variables with normal distribution were expressed as mean with standard deviation; data with skewed distribution were expressed as median with interquartile range (IQR 25–75%). Statistical comparisons between groups were obtained by *T*-Test or Mann-Whitney *U*-test for continuous variables according to the distribution. *p* value < 0.05 was considered statistically significant. Statistical analysis was performed using IBM SPSS Statistics 26.0 software (IBM Corporation, Armonk, NY, USA).

## Results

Thirty-four patients (15 females, 44.1%) affected by spinal dysraphism were enrolled in the period between May 2022 and April 2023. The median age of our sample was 18 years (12.0–22.2); 23 of them (67.6%) were affected by myelomeningocele (MMC) and 11 patients (32.4%) by lipomeningocele (LMC). The mean BMI was 24.3 (± 5.7) and it resulted pathological in 17 patients (50%). Twelve patients (35.3%) practiced physical activity with a median of 2 days (1.2–2.7) a week. The detailed characteristics of our sample are reported in Table 1.

**Table 1:** Clinical and demographics of study population

	Study population (*n* = 34)
Female, ***n*** (%)	15 (44.1)
Age (Y), median (IQR)	18.0 (12.0–22.2)
Diagnosis, ***n*** (%)	
MMC	23 (67.6)
LMC	11 (32.4)
BMI, mean (SD)	24.3 (± 5.7)
BMI pathological, ***n*** (%)	17 (50)
Physical activity, ***n*** (%)	12 (35.3)
Previous physical activity, ***n*** (%)	12 (54.5)*
Years of physical activity, median (IQR)	2 (1.2–2.0)
Days of physical activity per week, median (IQR)	2 (1.2–2.7)
Autonomous walking, ***n*** (%)	17 (50.0)
Mobility, ***n*** (%)	
Autonomous	17 (50.0)
Wheelchair	13 (38.2)
Walking aid	4 (11.8)
Hydrocephalus, ***n*** (%)	20 (58.8)
Oxybutynin, ***n*** (%)	30 (88.2)
Clean intermittent catheterization, ***n*** (%)	26 (76.5)
Barthel index, median (IQR)	87.5 (58.7–100.0)

*Evaluated in *n* = 22

All patients performed electrocardiography and echocardiography. One patient presented an altered ECG (right axis deviation) while no patients presented an altered echocardiography. In Tables 2 and 3 are detailed the results of those two exams.

**Table 2:** Resting electrocardiogram (ECG) characteristics

	Study population (*n* = 34)
Altered, *n* (%)	1 (2.9%)
Hear frequency, mean (SD)	85.5 (± 15.5)
PR interval, median (IQR)	128.0 (123.5–146.0)
QRS interval, mean (SD)	83.9 (± 8.4)
QT interval, mean (SD)	351.9 (± 26.6)
QTc interval (Bazett), mean (SD)	415.7 (± 14.7)

**Table 3:** Echocardiographic characteristics

	Study population (*n* = 34)
Pathological, *n* (%)	0
RVEDD, mean (SD) (right ventricular end-diastolic diameter)	17.5 (± 3.7)
LVEDD, median (IQR) (left ventricular end-diastolic diameter)	45.7 (44.7–50.0)
LVESD, mean (SD) (left ventricular end-systolic diameter)	30.1 (± 3.9)
IVSd, median (IQR)	7 (6.5–7.5)
PWd, median (IQR)	7 (6.5–7.5)
LVEF, median (IQR) (left ventricle ejection fraction)	58 (57–61)
Diastolic function, median (IQR)	1.4 (1.3–1.7)
Aortic bulb, mean (SD)	25.1 (± 3.0)
Aortic transvalvular speed, mean (SD)	1.2 (± 0.1)
Aortic arch median (IQR)	20 (16.9–21.1)
Descendent aortic blood speed, mean (SD)	1.3 (± 0.2)
Left atrium diameter, mean (SD)	29.1 (± 3.6)
Left atrium volume, mean (SD)	27.4 (± 9.9)
Indexed left atrium volume, mean (SD)	17.5 (± 4.8)

Twenty-seven patients (79,4%) performed a Cardiopulmonary Exercise Test (CPET) that resulted pathological in all of them. All the patients presented a pathological VO_2_ peak value, with a mean of 1191.0 (± 248.2); the mean VO_2_/Kg peak was 20.2 (± 4.4) and the mean VO_2_ peak % of the predict was 51.1 (± 7.8). The mean VO_2_/BCM ratio was 60.7 (± 17.3) and the VO_2_/FFM ratio was 29.3 (± 5.5) (Table 4).

**Table 4:** Cardiopulmonary Exercise Test (CPET) data

	Study population (*n* = 27)
Pathological, *n* (%)	27 (100)
Pathological VO_2_, *n* (%)	27 (100)
VO_2_ peak, mean (SD)	1191.0 (± 248.2)
VO_2_/Kg peak, mean (SD)	20.1 (± 4.4)
VO_2_ peak % predict, mean (SD)	51.1 (± 7.8)
VO_2_/BCM, mean (SD)	60.7 (± 17.3)
VO_2_/FFM, mean (SD)	29.3 (± 5.5)

The Body impedance assessment (BIA) has been performed in all patients and the results are reported in Table 5.

**Table 5:** Bioelectrical Impedance Analysis (BIA) data

	Study population (*n* = 33)
BCM, mean (SD)	19.7 (± 6.9)
FM, mean (SD)	19.0 (± 9.4)
FFM, mean (SD)	39.3 (± 10.9)
FFM%, mean (SD)	68.2 (± 10.7)
FM%, mean (SD)	31.8 (± 10.7)
TBW, mean (SD)	30.1 (± 8.2)

Thirty-one patients (91.2%) performed a DEXA scan. The mean BMC was 1.6 (± 0.4), the mean FM was 22.7 (± 10.2), the mean FFM was 35.4 (± 8.6), the mean FFM + BMC value was 37.0 (± 8.9) and the mean FM% was 36.6 (± 9.5).

For 27 patients the femoral *T*-score was reported and its mean value was − 1.3 (± 1.3); 11 patients (40.7%) were osteopenic and 5 patients (18.5%) were diagnosed as osteoporotic.

For 27 patients the lumbar-rachides *T*-score was collected and its mean value resulted of − 2.0 (± 1.8). Osteopenia was diagnosticated in 10 patients (37.0%) and osteoporosis in 8 patients (29.6%) (Table 6).

**Table 6:** Computerized Bone Mineralometry (CBM) and Dual Energy X-ray Absorptiometry (DEXA) data

	Study population (*n* = 31)
Femoral *T*-score, mean (SD)*	−1.3 (± 1.3)
Femoral *T*-score, *n* (%)*	
Osteopenia	11 (40.7)
Osteoporosis	5 (18.5)
Lumbar rachides *T*-score, mean (SD)*	−2.0 (± 1.8)
Lumbar rachides *T*-score, *n* (%)*	
Osteopenia	10 (37.0)
Osteoporosis	8 (29.6)
BMC, mean (SD)	1.6 (± 0.4)
FM, mean (SD)	22.7 (± 10.2)
FFM, mean (SD)	35.4 (± 8.6)
FFM + BMC, mean (SD)	37.0 (± 8.9)
FM%, mean (SD)	36.6 (± 9.5)

*Missing in *n* = 4

**Fig. 1:**
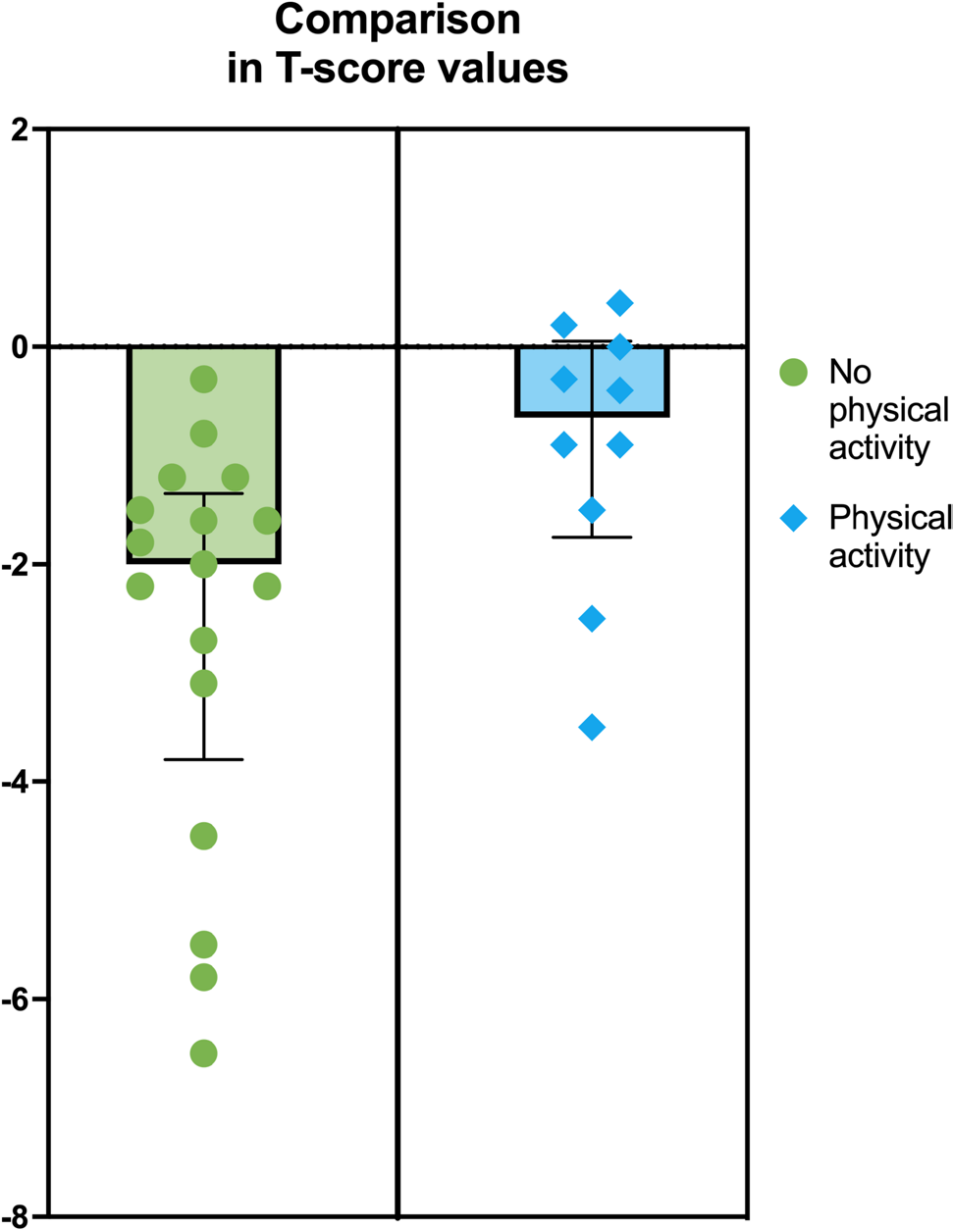
Comparison between *T*-score values of patients performing and those who do not perform physical activity

In Table 7 is reported a comparison between patients performing physical activity and patients not performing physical activity. The only statistically significative difference was the median lumbar *T*-Score, lower in the group not performing physical activity (− 2 (− 3.8 ÷  − 1.3) vs − 0.6 (− 1.7 ÷  − 0.05); *p* = 0.009) (Fig. 1).

**Table 7:** Comparison of patients performing and not performing physical activity

	Patients not performing physical activity (*n* = 22)	Patients performing physical activity (*n* = 12)	*p*
BMI, mean (SD)	24.6 (± 6.2)	23.9 (± 4.7)	0.75
Hear frequency, mean (SD)	87.6 (± 16.6)	81.6 (± 13.0)	0.29
VO_2_ peak, mean (SD)^a^	1110.3 (± 218.8)	1291.7 (± 254.5)	0.06
VO_2_/Kg peak, mean (SD)^a^	19.6 (± 4.1)	20.7 (± 4.8)	0.51
VO_2_ peak % predict, mean (SD)^a^	51.7 (± 9.0)	50.5 (± 6.4)	0.71
VO_2_/BCM, mean (SD)^a^	63.8 (± 16.7)	56.9 (± 17.9)	0.31
VO_2_/FFM, mean (SD)^a^	30.2 (± 5.6)	28.2 (± 5.3)	0.37
Femoral *T*-score, mean (SD)^b^	−1.5 (± 1.6)	−1.0 (± 0.8)	0.33
Lumbar *T*-score, median (IQR)^b^	−2 (−3.8 ÷ −1.3)	−0.6 (−1.7 ÷ −0.05)	0.009

^a^Missing in *n* = 7

^b^Missing in *n* = 7

## Discussion and conclusions

Patients with SB have several degrees of motor impairment which impacts on physical activity, depending, as reported, on the type and the level of neurological lesion.

In literature, there are few studies that evaluate the cardiological and respiratory profile and the bone mineralization status in patients with SB, especially about those who practice physical activity.

In a review published in 2018 [15], the bone fragility in patients with SB has been evaluated. The risk factors for the occurrence of fracture in SB include: high level of neurological involvement [16], hypomobility, the presence of hypercalciuria [17], and body mass index (BMI) values [18].

Computerized Bone Mineralometry has been the technique of choice for the measurement of bone mineral density (BMD) and, therefore, for the assessment of the presence of osteopenia and osteoporosis by detection of the *T*-score. A decrease in BMD is correlated with an increase in the neurological level of the lesion [19]; it has been observed a reduction of BMD values for all sites examined (lower extremities, lumbar region, and forearms), approximately 1 to 3 standard deviation (SD) scores below the mean of age and gender [20, 21].

According to this, in our study, we observed a compatible reduction of lumbar *T*-score (− 2 (− 3.8 ÷  − 1.3) vs − 0.6 (− 1.7 ÷  − 0.05); *p* = 0.009) (Fig. 1), but in literature is not reported a comparison between patients with SB who performing physical activity or not. The difference between the two groups highlights how physical activity could play a protective role in this category of patients.

In citated update are also reported the local risk factors that involve to the deficit of muscular strength, caused by neurological injury, that compromise BMD and increase the risk of occurrence of fractures [17]. The authors also suggested the role of systemic factors:

- Endocrine factors: the growth hormone (GH) deficiency has been documented in SB and is also related with a lower BMD than the healthy controls [22]; about obesity and metabolic syndrome, only children with ad higher-level lesions exhibited increased trunk fat [23].
- Central Nervous System (CNS): the link between leptin signaling, the hypothalamus, and the sympathetic regulation of bone turnover is recognized [24]. Hypothalamic dysregulation and moto-sensory neurological deficits can interfere with leptin secretion and with the bone turnover.
- Chronic Kidney Disease (CKD): congenital and acquired CKD may lead to a disordered regulation of mineral metabolism with subsequent alterations of bone remodeling and growth [25].
- Vitamin D role: patients with SB present vitamin D values significantly lower compared with healthy peers (vitamin D SB group: 14.6 ± 8.7 mg/dL; normal subjects: 35 ± 9.8 mg/dL; *p* < 0.001), so they need supplementation for a longer time to reduce the risk of fractures [26].

Authors have recognized the presence of other additional factors such as diet, environment, genetic, stress and strain on mechanical loads, and acidosis due to urinary diversion/augmentation cystoplasty.

Although with the inherent limitations of a small workload, our study is the first to report a significant difference in bone mineralization values between physically active and sedentary spina bifida patients. Further studies, on larger samples, are needed to better investigate this association.

Lastly, by means of the echocardiogram, congenital vascular anomalies affecting the main arterial vessels originating from the heart (aorta and pulmonary trunk), which could be suspected on the basis of the common embryological origin discussed above and which may also represent an absolute contraindication to the practice of leisure sport activities too, were ruled out in our patient population.

## Data Availability

The dataset used and analyzed during the current study is available from the corresponding author on reasonable request.
